# The potential impact of dietary choices on melanoma risk: an anti-inflammatory diet

**DOI:** 10.1186/s12263-024-00745-6

**Published:** 2024-05-23

**Authors:** Cristina Fortes, Simona Mastroeni, Lauretta Levati, Massimo Alotto, Francesco Ricci, Stefania D’Atri

**Affiliations:** 1 Epidemiology Unit, Istituto Dermopatico dell’Immacolata, IDI, Via dei Monti di Creta, 104, Roma, 00167 Italy; 2 National Centre for Disease Prevention and Health Promotion, Italian National Institute of Health (ISS), Rome, Italy; 3 Molecular Oncology Laboratory, IDI-IRCCS, Rome, Italy; 4 Melanoma Unit, IDI-IRCCS, Rome, Italy

## Abstract

The role of inflammation in the aetiology of cancer is recognized. However, no study yet examined the association between an anti-inflammatory diet and cutaneous melanoma and explored whether it could be modified by genetic variations in cyclooxygenase-2 (COX-2), a key enzyme in inflammation. A case-control study was conducted in the IDI-IRCCS hospital in Rome, Italy with 273 cases of primary cutaneous melanoma and 269 controls frequency matched to cases. Information on socio-demographic and pigmentary characteristics, medical history, sun exposure and dietary habits were collected for all subjects. The − 765G > C polymorphism was identified in DNA extracted from blood samples. An anti-inflammatory diet score was created. Logistic regression models were fitted to obtain odds ratios (ORs) and 95% confidence intervals (CIs). A high anti-inflammatory diet score (≥ 8 anti-inflammatory dietary items) was associated with a decreased risk of cutaneous melanoma (OR: 0.29; 95%CI: 0.17–0.49, P_trend_ < 0.0001) after adjusting for sex, age, education, number of common nevi, skin photo-type, solar lentigines and sunburns in childhood. COX-2 -765 G > C polymorphism was not an independent risk factor for cutaneous melanoma. Although interaction between − 765G > C genotypes and anti-inflammatory diet score was not statistically significant (*p* = 0.25), when stratified by -765 G > C genotypes the effect of the anti-inflammatory diet was slightly more pronounced for participants carrying – 765GG (OR: 0.17; 95%CI: 0.06–0.47, P_trend_ < 0.001). Our study findings suggest that adherence to an anti-inflammatory diet is associated with a decreased risk of developing cutaneous melanoma. These results suggest the potential impact of dietary choices on melanoma risk.

## Introduction

Cutaneous melanoma is an aggressive cancer that tends to metastasize, and its incidence is increasing globally [1, 2]. In the last decades, cutaneous melanoma worldwide incidence reached 325.000 new cases per year [1]. In Italy, cutaneous melanoma is the third most common cancer in young age (< 50 years) and in 2020 its incidence was 14.900 new cases [3]. In terms of incidence, estimated projections advocate that cutaneous melanoma will become the second most common cancer worldwide [4]. Despite, recent advances in cutaneous melanoma treatment, its burden is rather high. In order to curb the progressive trend in incidence of cutaneous melanoma, it is crucial to gain a better understanding of risk factors by integrating both genetic and environmental data. The most important risk factors for cutaneous melanoma include pigmentary characteristics, family history of melanoma, presence of freckles, sun exposure and reactions to sun exposure [5–7]. Amid the multifaceted landscape of potential factors contributing to cancer aetiology, inflammation has gained increasing recognition [8]. Chronic Inflammation is known, indeed, to influences the development of cancer by stimulating cancer cell proliferation, promoting angiogenesis and inhibiting apoptosis [9].

Epidemiological studies have indicated that a pro-inflammatory diet is associated with an increased risk of cancer [10, 11]. Conversely, some dietary components rich in anti-inflammatory properties, could potentially play a protective role in the aetiology of cancer including melanoma [12]. Polyphenols such as resveratrol, are one of these dietary components. They have been shown to have a direct impact on immune modulation and on the prevention of oxidative stress [13]. Despite the evidence, a critical gap exists in the literature, as no study has examined whether adherence to an anti-inflammatory diet modifies the risk of developing cutaneous melanoma. Intriguingly, the enzyme cyclooxygenase-2 (COX-2), known for its role in inflammation emerges as a potential genetic modifier in this context.

COX-2 is an inducible enzyme involved in the synthesis of prostaglandins, bioactive lipids that play a key role in inflammatory responses [14, 15]. COX-2 expression can be induced by cytokines, growth factors and various proinflammatory stimuli and is regulated at both transcriptional and post-transcriptional level [14, 16]. COX-2 is over-expressed in many cancers including melanoma [17, 18] and has been linked to angiogenesis, inhibition of apoptosis, stimulation of invasion and suppression of immune responses [9, 17]. Accordingly, non-steroidal anti-inflammatory drugs (NSAIDs) that inhibit COX-2 have been associated with a reduced risk of certain cancers [19, 20].

Moreover, several polymorphisms have been identified in the COX-2 gene and some of them have been shown to affect basal expression of the gene and/or its induction in response to specific stimuli and to be associated with increased cancer risk [18, 21–26].

The role of different COX-2-765G > C genotypes in cancer has been investigated in many epidemiological studies but with conflicting findings. It has been suggested that the − 765 C allele has a decreased promoter activity with low PGE2 production [23]. However, epidemiological studies suggest that − 765 C allele is a risk factor for some cancers such as oesophageal, pancreatic and gastric cancer, as well as for leukaemia [24–26]. In contrast, a lower risk of developing basal cell carcinoma was associated with the − 765 C allele in patients undergoing organ transplantation before 50 years of age [18]. The interplay between COX-2-765G polymorphism, anti-inflammatory dietary patterns, and the risk of cutaneous melanoma remains a largely unexplored area.

Given the evidence, the aim of the present study was to bridge this critical gap by investigating the association between adherence to an anti-inflammatory diet, -765G > C polymorphism and the risk of cutaneous melanoma.

## Methods

This study was conducted within a case-control study conducted between 2001 and 2005 that aimed to investigate new environmental and nutritional risks factors for cutaneous melanoma and explore gene-environmental interactions. It was conducted at a referral hospital for skin diseases (IDI-San Carlo) in the Lazio Region of Italy (*N* = 542). Cases (*N* = 273) and controls (*N* = 269) were Caucasian origin aged 18 years or more and resident in Lazio. All cases had a new histologically confirmed diagnosis of primary malignant cutaneous melanoma. Controls were identified among patients admitted to Orthopaedics, General Surgery, Vascular Surgery, General Medicine and Ophthalmology from the same hospital and geographic area (Lazio region) and frequency matched to cases by sex (1:1) and age (in 5-year age strata). Skin diseases and cancer were excluded from the control series and a balance between diagnoses was maintained while enrolling controls. The main diagnoses were as follow: injury, benign neoplasms, diseases of the genitourinary system, diseases of the musculoskeletal system and diseases of the circulatory system.

The response rate i.e. percentage of cases and controls that accepted to participate in the study and completed all questionnaires and had a dermatological visit was 94.7% and 92.1% for cases and controls respectively. Out of these 306 cases and 309 controls, 273 cases (89.2%) and 269 (87.05%) also donated blood.

The study protocol was approved by the Institutional Review Board of the Ethics Committee of the Istituto Dermopatico Dell’Immacolata, IDI-IRCCS (CE, No. 106), Rome, Italy and it was conducted according to the Declaration of Helsinki. After obtaining informed consent, a structured questionnaire was administrated to participants, and a clinical visit to identify and register pigmented lesions was conducted by two trained dermatologists. Researchers that administrated the questionnaire were blinded to the status (cases or controls) of the subjects. The questionnaire included data on sociodemographic characteristics, medical history, smoking habits, phenotypic traits (skin type, skin, hair and eye colour), family history of skin cancer, life time sunlight exposure, sunburn history and diet. The presence of chronic diseases (diabetes, cardiovascular diseases, asthma, rhinitis, atopic dermatitis, bronchitis, lupus, arthritis) was recorded. A standard protocol was used in our study to identify and record pigmented lesions [27]. The number of nevi (≥ 2 mm) over the entire skin surface were recorded and then classified as low (0–59) and high (≥ 60). Other skin and individual characteristics, such as freckles and solar lentigines and information on history of skin cancer, were also recorded. Solar lentigines were categorized as: none, few (one body area), moderate (two body areas), or many (two body areas or more). The Fitzpatrick system was used to determine skin photo-type (burning and tanning tendency) [28]. Hair colour was classified as fair/blonde/red, light brown, dark brown and black. During the interview, a skin and hair colour chart were used for defining skin, hair and eye colour. Smoking status was classified as never smokers, ex-smokers and current smokers. Smokers were considered as such if they smoked at least one cigarette daily for six months. Ex-smokers were defined as those who had stopped smoking at least 1 year before the melanoma diagnosis. Food frequency questionnaires, that are widely used instruments in Epidemiology, usually refer to a single year but they are representative of dietary intake over many years [29]. A validated food frequency questionnaire that included also fresh herbs consumption was used to evaluate dietary habits [30, 31]. Trained personnel administrated the food frequency questionnaire to participants and asked them to report their average dietary intake in the past 12 months (before diseases diagnosis). Neither the personnel in charge of the questionnaire administration nor the cases and controls were aware of the hypothesis of the study. A seven-point scale was used to classify the frequency intake of foods as follows: never, less than monthly, less than weekly, 1–2 time weekly, 3–4 weekly, 5–7 weekly and daily.

Anti-inflammatory foods are getting increasing attention because the potential benefits to human health but as far as we know there is no “anti-inflammatory diet score” yet published in the scientific literature. In our study we used the publication of Shivappa and colleagues (2014) as a resource to define foods as “anti-inflammatory”. Shivappa and colleagues (2014) classified food items or nutrients using biomarkers of inflammation. For instance, an anti-inflammatory food item was defined as the one that decreased IL-1β, IL-6, TNF-α and C-reactive protein [32].

Food items classified as anti-inflammatory were as follows: green leafy vegetables, salad, vegetables in general, citrus fruits, fruits in general, nuts, exclusive use of olive oil for cooking and dressing, fresh rosemary, fresh salvia, coffee, fish rich in n-3 fatty acids. High consumption of an anti-inflammatory food item was scored (1) and low consumption or no consumption of that food item was scored (0). High consumption was defined as follows: green leafy vegetables and salad (≥ 3 times/week); citrus fruits and other vegetables (≥ 5 times/week), fruits in general and coffee (daily or more), nuts and fish rich in n-3 fatty acids (weekly and more), exclusive use of olive oil for cooking and dressing (yes/no), regular use of fresh rosemary (yes/no) and fresh salvia (yes/no). Foods that were not defined as anti-inflammatory (e.g. pro-inflammatory foods or neither anti-inflammatory or pro-inflammatory) were scored as (0). In this way all foods consumed by the subjects were taken into consideration. The score ranged from 0 to 11 and the higher the score the better the compliance with an anti-inflammatory diet. Categories of the anti-inflammatory score were based on the terciles of the controls’ distribution. Three categories of the anti-inflammatory score were created (low: ≤ 5; medium: 6–7; high: ≥ 8).

Physical measurements of body weight and height were used to calculate Body mass index (BMI) BMI was categorized in three classes (≤ 24.9; 25.0-29.9; ≥30 kg/m^2^). Information on the use of non-steroidal anti-inflammatory drugs (NSAIDs) and the presence of chronic diseases were collected from medical records at the beginning of the study period. The use of NSAIDs was classified as yes/no. Participants were considered as consumers if they used NSAIDS for one or more consecutive periods of 7 days. Lifetime sun exposure was the sum of the average hours outdoors during lifetime. Lifetime sun exposure was classified into terciles (low: ≤ 26; medium: 27–36; high: ≥37 h) based on the controls’ distribution. Due to the limited number of individuals homozygous for the allele C (*N* = 17), a variable was created that combined subjects carrying either 1 or 2 alleles C. Subjects with the − 765GC and − 765CC genotypes were pooled together.

### COX-2 − 765G > C genotyping

Genomic DNA was extracted from peripheral blood leukocytes using DNeasy Blood & Tissue kit (QIAGEN, Valencia, CA, USA). The analysis of COX-2 -765G > C genotypes was performed by the PCR-based restriction fragment length polymorphism (PCR-RFLP) assay. The promoter region surrounding the − 765G > C polymorphism site was amplified using the following primers: Forward 5’-CATTAACTATTTACAGGGTAACTGCTT-3’, reverse 5’-TGCAGCACATACATACATAGCTTTT-3’.

Amplification was performed in 25 µL PCR reaction volume containing 50 ng genomic DNA, 0.2 µM of each dNTP, 12.5 pmol of each primer and 1.25 U Taq Polymerase in 1X PCR buffer.

Thermal cycling conditions included an initial denaturation step at 94 °C for 5 min followed by 35 cycles of 94 °C for 30 s (denaturation), 56 °C for 30 s (annealing), 72 °C for 30 s (extension), with a final extension step of 72 °C for 10 min. Primers generated a DNA fragment of 228 bp in length which was purified with Wizard® SV Gel and PCR clean up system kit (Promega Corporation, Madison, USA) and then digested with Fau I (New England Biolabs, Beverly, MA, USA) restriction endonuclease at 55 °C for 4 h. The enzyme cut the PCR product into fragments of 161 bp and 67 bp if G allele was present. Genotypes were determined by restriction fragments size as follows: two fragments of 161 and 67 bp indicated the homozygous GG genotype, an uncut fragment of 228 bp indicated the homozygous CC genotype, three fragments of 228 bp, 161 bp and 67 bp indicated the presence of heterozygous GC genotype. The digestion products were separated on 2% agarose gel and visualized by GelRed™ Nucleic Acid Gel Stain (Biotium Inc., Hayward, CA, USA). The results of RFLP analysis were further confirmed by direct sequencing in randomly selected samples, with 100% concurrence rate.

### Statistical analysis

Cases and controls were compared using the Chi-square test for categorical variables and the Mann–Whitney U test for continuous variables. We used odds ratios (ORs) with 95% confidence intervals (CIs) to investigate the association between the anti-inflammatory diet score and cutaneous melanoma risk. We first conducted an analysis for all known risk factors for cutaneous melanoma (e.g. sun exposure, phenotypic traits, number of nevi). We avoided keeping variables in the model that were highly associated and did not contribute to the fit of the model such as pigmentary characteristics and skin photo-type. The following variables were considered in the multivariate logistic regression models as potential confounders: sex, age, education, BMI, skin photo-type, solar lentigines, number of nevi and sunburn episodes in childhood. The likelihood test ratio test was used to decide whether to keep each variable in the model. Only those variables that were statistically significant and made contributions to the model were included (*p* < 0.05). To test for linear trend, we modelled the anti-inflammatory score as an ordinal variable. The likelihood test ratio test was also used to test for interaction between COX-2 genotypes and anti-inflammatory diet score.

To test the robustness of the results we ran all models controlling, one at a time in the model, for the presence of chronic diseases (e.g. diabetes, cardiovascular diseases, asthma, rhinitis, atopic dermatitis, bronchitis, lupus, arthritis), BMI, smoking, NSAID drugs use and the use of only aspirin. BMI was introduced in the model as an indirect indicator of energy intake. Subgroup analyses were conducted by COX-2 genotypes. All analyses were performed using the statistical software package PC-STATA (STATA, release 15 (College Station, TX: StataCorp LCC).

## Results

A total of 542 subjects were interviewed and had a full skin examination (273 cases, 46.1% males and 53.9% females; 269 controls 43.5% males and 56.5% females). The most frequent histological type of melanoma was superficial spreading (75.10%) followed by nodular type (11.0%). The mean age of the cases and controls was 53.7 years (SD 15.3 years) and 51.9 years (SD 15.6 years), respectively (*p*-value = 0.205). Out of the 542 subjects who participated in the study, 303 donated blood. Table 1 illustrates the association between socio-demographic, pigmentary characteristics and − 765G > C polymorphism and cutaneous melanoma. Cases were more highly educated than controls (OR: 2.87; 95% CI:1.52–5.40), had lighter hair (OR: 4.31; 95% CI: 2.46–7.54) and fair skin colour (OR: 3.79; 95% CI: 2.50–5.74), a higher number of freckles (OR: 3.52; 95% CI: 2.33–5.31) more common nevi (OR: 5.41; 95% CI: 3.42–8.54) and solar lentigines (OR: 3.60; 95% CI: 2.33–5.55). Genotype frequencies of − 765G > C in our population were as follows: CC (5.6%), CG (33.7%), GG (60.7%) (Table 1). No association was found between − 765G > C polymorphism and cutaneous melanoma. When subjects with the − 765GC and − 765CC genotype were pooled together, no association was found (CC + GC versus GG; OR: 0.85; 95% CI: 0.52–1.38). The mean anti-inflammatory diet score was higher in controls (6.2, SD = 2.1) in comparison to cases (5.5, SD = 1.9, *p* = 0.001).

**Table 1:** Characteristics of the subjects participating in the study by status

	All					
		cases	controls			
Characteristics, N. (%)	(N^a^=542)	(N^a^=273)	(N^a^=269)	*P*value^b^	OR(95%CI)	*P*value
Sex						
males	243 (44.8)	126 (46.1)	117 (43.5)			
females	299 (55.2)	147 (53.9)	152 (56.5)	0.534		
Age, y						
mean (SD)	52.8 (15.5)	53.7 (15.3)	51.9 (15.6)			
median (IQR)	53.6 (40.1–65.3)	54.1 (40.6–66.0)	52.6 (39.3–64.6)	0.205^c^		
Education (yr)						
< 8	105 (19.4)	46 (16.9)	59 (21.9)		1	
8–13	345 (63.8)	166 (61.0)	179 (66.5)		1.43 (0.87–2.35)	0.160
> 13	91 (16.8)	60 (22.1)	31 (11.5)	0.003	2.87 (1.52–5.40)	0.001
Hair colour						
black/dark brown	292 (53.9)	113 (41.4)	179 (66.5)		1	
light brown	173 (31.9)	105 (38.5)	68 (25.3)		2.62 (1.76–3.90)	< 0.0001
fair/blond/red	77 (14.2)	55 (20.1)	22 (8.2)	< 0.0001	4.31 (2.46–7.54)	< 0.0001
Skin photo-type^d^						
III-IV	247 (45.7)	92 (33.8)	155 (57.8)		1	
I-II	293 (54.3)	180 (66.2)	113 (42.2)	< 0.0001	2.80 (1.95–4.03)	< 0.0001
Skin colour						
dark	155 (28.8)	45 (16.5)	110 (41.3)		1	
fair	384 (71.2)	228 (83.5)	156 (58.7)	< 0.0001	3.79 (2.50–5.74)	< 0.0001
Presence of freckles						
No	363 (69.5)	150 (57.7)	213 (81.3)		1	
Yes	159 (30.5)	110 (42.3)	49 (18.7)	< 0.0001	3.52 (2.33–5.31)	< 0.0001
Common nevi (n)						
0–59	398 (73.4)	164 (60.1)	234 (87.0)		1	
≥ 60	144 (26.6)	109 (39.9)	35 (13.0)	< 0.0001	5.41 (3.42–8.54)	< 0.0001
Solar lentigines						
none/few/moderate	157 (29.2)	47 (17.4)	110 (41.2)		1	
high	380 (70.8)	223 (82.6)	157 (58.8)	< 0.0001	3.60 (2.33–5.55)	< 0.0001
Family history of skin cancer						
No	516 (96.1)	259 (95.6)	257 (96.6)		1	
Yes	21 (3.9)	12 (4.4)	9 (3.4)	0.532	1.32 (0.54–3.25)	0.539
Presence of chronic disease (excluding CVDs)						
No	386 (71.2)	197 (72.2)	189 (70.3)		1	
Yes	156 (28.8)	76 (27.8)	80 (29.7)	0.625	0.93 (0.64–1.36)	0.703
Presence of chronic disease (including CVDs)						
No	365 (67.3)	182 (66.7)	183 (68.0)		1	
Yes	177 (32.7)	91 (33.3)	86 (32.0)	0.735	1.08 (0.75–1.56)	0.673
-765 G > C genotypes						
-765GG	184 (60.7)	102 (61.8)	82 (59.4)		1	
-765GC	102 (33.7)	55 (33.3)	47 (34.1)		0.89 (0.53–1.47)	0.637
-765CC	17 (5.6)	8 (4.9)	9 (6.5)	0.795	0.67 (0.24–1.89)	0.445

*Abbreviation*: SD, Standard Deviation; IQR, Interquartile Range; CVD, cardiovascular diseases

a: Totals may vary because of missing value; b: χ2 test; c: Mann-Whitney U test; d: I-always burns, never tans; II-often burns, tans minimally; III-rarely burns, tans well; IV-never burns, tans profusely.

Table 2 shows the association between sun exposure, smoking, BMI, anti-inflammatory diet score, the use of COX-2 inhibitor drugs and cutaneous melanoma. Sunburn in childhood was associated with an increased risk of cutaneous melanoma (OR: 2.50; 95% CI:1.64–3.79). Subjects ranked with a high anti-inflammatory diet score (≥ 8) had the risk of melanoma three times lower in comparison to subjects ranked with a low score (≤ 5) (OR: 0.35; 95%CI: 0.22–0.55, *p* < 0.0001). No interaction was seen between − 765G > C genotypes and anti-inflammatory diet score (*p* = 0.25).

**Table 2:** Sun exposure, life-style habits and use of Cox-2 inhibitor drugs of the subjects participating in the study by status

	All						
		Cases	Controls				
Characteristics, N. (%)	(N^a^=542)	(N^a^=273)	(N^a^=269)		*P*value^b^	OR(95%CI)	*P*value
Lifetime sun exposure (hours)							
low (≤ 26)	181 (35.4)	93 (36.6)	88 (34.1)			1	
medium (27–36)	162 (31.6)	73 (28.7)	89 (34.5)			0.76 (0.49–1.17)	0.210
high (≥ 37)	169 (33.0)	88 (34.7)	81 (31.4)		0.373	0.99 (0.63–1.54)	0.95
Sunburns in childhood							
no	297 (66.3)	121 (56.5)	176 (75.2)			1	
yes	151 (33.7)	93 (43.5)	58 (24.8)		< 0.0001	2.50 (1.64–3.79)	< 0.0001
BMI							
≤ 24.9	282 (52.2)	150 (55.1)	132 (49.3)			1	
25.0-29.9	195 (36.1)	91 (33.5)	104 (38.8)			0.68 (0.45–1.01)	0.056
≥ 30	63 (11.7)	31 (11.4)	32 (11.9)		0.368	0.76 (0.43–1.34)	0.343
Smoking status							
never smokers	252 (46.6)	126 (46.3)	126 (46.8)			1	
current smokers	193 (35.7)	94 (34.6)	99 (36.8)			0.98 (0.66–1.45)	0.916
ex smokers	96 (17.7)	52 (19.1)	44 (16.4)		0.677	1.12 (0.68–1.85)	0.648
Anti-inflammatory diet Score							
low (T1, ≤ 5)	244 (45.0)	137 (50.2)	107 (39.8)			1	
medium (T2, 6–7)	173 (31.9)	95 (34.8)	78 (29.0)			0.87 (0.58–1.30)	0.490
high (T3, ≥ 8)	125 (23.1)	41 (15.0)	84 (31.2)		< 0.0001	0.35 (0.22–0.55)	< 0.0001
Use of Cox-2 inhibitor drugs							
no	490 (90.4)	250 (91.6)	240 (89.2)			1	
Yes	52 (9.6)	23 (8.4)	29 (10.8)		0.352	0.68 (0.37–1.23)	0.201
Use of aspirin							
No	513 (94.7)	254 (93.0)	259 (96.3)			1	
Yes	29 (5.3)	19 (7.0)	10 (3.7)		0.094	1.89 (0.83–4.29)	0.129

*Abbreviation*: SD, Standard Deviation; IQR, Interquartile Range

a: Totals may vary because of missing value; b: χ2 test.

Table 3 shows the results of multivariate models. After adjusting for sex, age, education, number of nevi, skin photo-type, solar lentigines and sunburns in childhood, the effect persisted (OR: 0.29; 95%CI: 0.17–0.50, p-trend < 0.0001). After introducing smoking, BMI and COX-2 inhibitor drugs the risk estimate did not change. In all multivariable models, common nevi, skin photo-type, solar lentigines and sunburns in childhood remained associated with melanoma.

**Table 3:** Association between Anti-inflammatory Dietary Score and cutaneous melanoma: multivariate analysis

		Anti-inflammatory Diet score			
		low (T1, ≤ 5)	medium (T2, 6–7)	high (T3, ≥ 8)	
Model	Variables	(reference)	ORadj(95%CI)	ORadj(95%CI)	*P*_trend_^*a*^
A	sex, age, education, number of nevi, skin photo-type, solar lentigines and sunburns in childhood	1	0.83 (0.53–1.31)	0.29 (0.17–0.50)	< 0.0001
B	model A + Body Mass Index	1	0.84 (0.53–1.32)	0.29 (0.17–0.50)	< 0.0001
C	model A + use of Cox-2 inhibitor drugs	1	0.84 (0.53–1.33)	0.29 (0.17–0.49)	< 0.0001
D	model A + use of aspirin	1	0.83 (0.52–1.30)	0.30 (0.17–0.51)	< 0.0001
E	model A+ -765 G > C genotypes	1	0.67 (0.36–1.27)	0.25 (0.12–0.52)	< 0.0001
F	model A + smoking	1	0.82 (0.52–1.31)	0.29 (0.17–0.50)	< 0.0001
G	model A + chronic disease (excluding CVDs)	1	0.83 (0.53–1.32)	0.29 (0.17–0.50)	< 0.0001
H	model A + chronic disease (including CVDs)	1	0.83 (0.53–1.31)	0.29 (0.17–0.50)	< 0.0001

T, tercile; OR, odds ratio; CI, confidence intervals; CVD, cardiovascular diseases

a: test for trend (Wald Test)

In a further analysis, we stratified for − 765G > C genotypes and the effect of the anti- inflammatory diet score remained. For subjects carrying the GG allele (OR: 0.17; 95%CI: 0.06–0.47, P_trend_ < 0.001) the effect was more pronounced than for subjects carrying the C allele (OR: 0.21; 95%CI: 0.06–0.78) (data not shown).

## Discussion

It is widely accepted that both genetic and environmental factors have a role in the genesis of melanoma [33]. Pigmentary characteristics, family history of melanoma, presence of freckles, skin colour and skin reactivity to sun are examples of non-modifiable risk factors for melanoma while sun exposure and sun bed use are modifiable risk factors [5–7]. Amid the multifaceted landscape of potential factors contributing to cancer aetiology, inflammation has gained increasing recognition [8]. Chronic inflammation has been suggested to impact the development of cancer by stimulating cancer cell proliferation, promoting angiogenesis and inhibiting apoptosis [9]. Epidemiological studies show that anti-inflammatory foods may play a protective role in the aetiology of cancer [12]. However, until now, no study investigated whether adopting an anti-inflammatory diet could modify the risk of cutaneous melanoma. Our study addressing this gap, shows that individuals with a high anti-inflammatory diet score, have threefold lower risk of melanoma compared to those with a low anti-inflammatory diet score, even after controlling for all possible confounding factors.

The observed effect of the anti-inflammatory diet on cutaneous melanoma in our study suggests potential mechanisms at play. One plausible explanation is the modulation of the systemic immune response by natural phytochemicals present in anti-inflammatory foods [34]. Another possible explanation could be through the modulation of the host gut microbiome. Indeed, previous research has indicated that individuals with a diet rich in anti-inflammatory foods, including nuts, vegetables, fruits, fish with high n-3 fatty acids, harbour in the gastrointestinal tract microorganisms (e.g. Roseburia hominis, Faecalibacterium) with anti-inflammatory effects, influencing the systemic immune response [35].

The role of different COX-2-765G > C genotypes in cancer is not yet clear. Epidemiological studies suggest that − 765 C allele is a risk factor for some cancers (e.g. oesophageal, pancreatic and gastric cancer, as well as for leukaemia) [24–26] but not for all [18].

Our study showed that COX-2-765G > C polymorphism is not an independent risk factor for cutaneous melanoma. This aligns with previous studies that similarly reported no link between this polymorphism and skin cancer, including melanoma [36, 37]. In a study conducted in patients subjected to renal transplantation no association was found between COX-2-765G > C polymorphism and the risk of developing skin cancer [36]. In another study the − 765 C allele was associated with an increased risk of melanoma in an unadjusted model but not after correcting for multiple testing [37].

Importantly, we also showed, for the first time, that COX-2-765G > C polymorphism does not modify the role of the anti-inflammatory diet on cutaneous melanoma development. Interestingly, our study revealed that the effect of the anti-inflammatory diet was slightly more pronounced for subjects carrying the GG allele. The reason behind this observation could be attributed to the higher number of individuals with this genotype. The GG genotype is associated with a decreased risk for developing some cancers [26] and it is associated with low levels of IL-6 amongst patients with coronary artery diseases [38].

The strength of our study lies in its methodology, combining individual clinical and nutritional data alongside the COX-2 polymorphism analysis. The high response rate of cases and controls minimizes potential selection bias and the observed dose-response relationship between anti-inflammatory diet score and melanoma add robustness to our findings.

However, there are also limitations in the study that merit discussions such as the presence of recall bias that is intrinsic to all observational studies. The influence of the current diet on recall of the diet may lead to bias when the eating habits of the cases have changed as a result of diagnosis. To mitigate this, in our study we included only incident cases. Additionally, our study is not large enough to draw firm conclusions regarding the association between COX-2-765G > C polymorphism and cutaneous melanoma. Additional studies with larger sample size would further confirm the findings.

Results from genome-wide association studies (GWAS) show that cutaneous melanoma susceptibility is most likely related to a polygenic inheritance pattern [39, 40]. Our study focused on a single susceptibility polymorphism and this could be a limitation. However, the objective of our study was not to identify an “at risk population” for cutaneous melanoma for future screening, but to investigate the impact of an anti-inflammatory diet on melanoma risk and assess whether the COX-2-765G > C polymorphism could act as a genetic modifier of melanoma risk associated with dietary habits.

In conclusion, our study findings suggest that adherence to an anti-inflammatory diet is associated with a decreased risk of developing cutaneous melanoma. These results not only suggest the potential impact of dietary choices on melanoma risk but also emphasizes the need for further research to delve deeper into the intricate interplay between genetics, diet and melanoma development.

## Data Availability

Data is provided within the manuscript.
